# Fundus Photography-Based Distribution of Retinal Hemorrhages in Newborns: Implications for Underlying Mechanisms

**DOI:** 10.3390/jpm15010038

**Published:** 2025-01-19

**Authors:** Gwon Hui Jo, Mi Young Choi, Kibum Lee, Kyung Tae Kim, Dong Yoon Kim, Ju Byung Chae, Eoi Jong Seo

**Affiliations:** 1 Department of Ophthalmology, Chungbuk National University Hospital, Chungbuk National University College of Medicine, Cheongju 28644, Republic of Korea; rjsgl6117@naver.com (G.H.J.); mychoi@chungbuk.ac.kr (M.Y.C.); ki4202009@hanmail.net (K.L.); kt-jins@hanmail.net (K.T.K.); 2 Seoul Top Eye Center, Cheongju 06083, Republic of Korea; umlover9@gmail.com (D.Y.K.); cjbmed@naver.com (J.B.C.)

**Keywords:** major vascular arcade, newborn, retinal capillary area, retinal hemorrhage

## Abstract

**Introduction:** The aim of this study was to investigate the locational distribution and potential mechanisms of retinal hemorrhages in newborns using fundus photography. **Methods:** A retrospective analysis of 98 consecutive newborns with retinal hemorrhages in at least one eye and 30 control newborns without retinal hemorrhages after uneventful delivery was conducted. Retinal hemorrhages were diagnosed and characterized using fundus photography and indirect ophthalmoscopy. The location, grade, and features of the hemorrhages were analyzed, alongside their association with delivery mode. Visual function was assessed at a mean follow-up of 7.8 months to evaluate the long-term implications. **Results:** Retinal hemorrhages were significantly associated with normal spontaneous vaginal delivery (NSVD) compared to cesarean section (*p* = 0.004). Bilateral involvement was observed in 87.8% of cases, with hemorrhages predominantly located around the major vascular arcade (MVA) and near the optic disc. Higher grades of hemorrhages were linked to increased involvement of the macula and retinal capillary area (RCA) (*p* < 0.001). All hemorrhages resolved spontaneously within 45.6 ± 15.9 days. No significant differences in refractive errors or strabismus development were identified between the hemorrhage and control groups at follow-up. **Conclusions**: Neonatal retinal hemorrhages are commonly observed near the MVA and optic disc, with greater severity associated with macular and RCA involvement. These findings, along with the significant association with NSVD, support a mechanism related to elevated central venous pressure. Retinal hemorrhages resolve spontaneously without impacting refractive error or strabismus development in the short term follow-up.

## 1. Introduction

Retinal hemorrhages can be observed in healthy newborns and are presumably associated with the birth process. The incidence of retinal hemorrhage in newborns varies widely between 2.6% and 50.0% [1,2,3,4], and it usually resolves spontaneously within two weeks [5]. Birth-related retinal hemorrhages in newborns do not directly cause visual or neurologic deficits [6], but reports on them are inconclusive. The known risk factors of retinal hemorrhages in newborns are the use of forceps and vacuum-assisted delivery [1]. Delivery by cesarean section is less likely than normal spontaneous vaginal delivery (NSVD) to result in retinal hemorrhages in newborns [1]. Little is known about the long-term consequences of retinal hemorrhages in newborns. Vision development is initiated after birth; therefore, the obstruction of the visual axis by a hemorrhage for a given period may induce amblyopia, which is a leading cause of monocular visual impairment among children [7].

Fundus photography facilitates immediate visualization and real-time recording of the retina. It has been useful in recording and documenting fundus abnormalities, especially in infants, for the diagnoses of retinal hemorrhage or retinopathy of prematurity in newborns [8]. Several studies on retinal hemorrhages in newborns have relied on the findings of manual fundus examination with direct or indirect ophthalmoscopes [5,9,10,11]. However, the characteristics of retinal hemorrhages in newborns have rarely been investigated.

This study aimed to investigate the severity, location, and characteristics of retinal hemorrhages in newborns using fundus photography. We also conducted a longitudinal analysis for up to 7.8 months to assess those who had macular hemorrhages at birth for strabismus and refractive errors. This study may help elucidate the mechanism underlying retinal hemorrhages in newborns and identify their implications on visual development.

## 2. Methods

We reviewed 196 eyes of 98 infants diagnosed with newborn retinal hemorrhages at Chungbuk National University Hospital between October 2018 and March 2021. Both eyes of each infant were included in the analysis if retinal hemorrhage was present in at least one eye. As a control group, 30 infants without any retinal hemorrhages in both eyes were also included independently. The study was conducted in accordance with the Declaration of Helsinki, and approved by the Institutional Review Board of the Chungbuk National University Hospital (protocol code: 2022-11-016; date of approval: 1 December 2022). The requirement for informed consent from participants was waived because of the retrospective nature of this study.

All infants underwent fundus photography (Clarity Medical Systems, Pleasanton, CA, USA) during the first week of birth (Exam 1). The fundus photographs were then read by ophthalmologists. Infants who had retinal hemorrhages in the fundus photographs in Exam 1 were diagnosed with newborn retinal hemorrhage, and included. Infants who did not show any retinal hemorrhages in both eyes in Exam 1 were classified into the control group. Infants with images of poor quality, which included 144 eyes of 72 infants, were also excluded. Images that exhibited blurring, dark fades, or were out of focus over more than 30% of the retina were classified as poor-quality images. Additionally, images where the center was not fixated on the fovea were also excluded.

Infants with newborn retinal hemorrhage underwent a secondary dilated fundus examination about a month later by a retinal specialist (Exam 2) using indirect ophthalmoscopy to confirm the improvement in or resolution of the hemorrhage. Afterwards, it was recommended that they undergo a follow-up examination (Exam 3) in approximately six months. Infants in the control group who showed no retinal hemorrhage in Exam 1 did not undergo Exam 2 and were followed up in Exam 3. Exam 3 was performed by a single pediatric ophthalmology specialist when the patients re-visited the clinic after about six months. The assessment of visual function was carried out by evaluating the patient’s ability to fixate and follow a light source, as well as by performing the cover/uncover test and the alternate cover test at both near (30 cm) and far (5 m) distances. While more objective tests, such as optokinetic drum, electroretinogram, and visual evoked potential, are preferred for evaluating visual function in infants under six months, these were not performed due to the retrospective nature of this study. As this study was conducted in the context of routine clinical practice for screening purposes, the fix-and-follow test and the cover/uncover test were employed as simple, practical methods for assessing fixation and alignment. The examiner performed noncycloplegic manifest refraction using retinoscopy in a dark room, while an assistant produced noise with a toy to keep the infants fixated on the toy located at a distance of 5 m. Despite this effort, 35 out of 98 infants (35.7%) were unable to complete the test. In contrast, all infants cooperated well with the near test. The infants who did not cooperate with refraction were excluded from this study. Cycloplegic refraction was not performed in this study due to its retrospective nature, as examinations were conducted as part of routine clinical practice rather than for research purposes. Tropicamide, while generally safe, does not provide a complete cycloplegic effect when used alone, and stronger agents such as atropine or cyclopentolate pose a higher risk of systemic side effects in neonates [12]. Therefore, non-cycloplegic refraction was selected as a safer alternative, and it was deemed sufficient for detecting significant refractive errors in infants under six months of age.

Demographic data, including obstetric information such as sex, gestational age, birth weight, and method of delivery, were collected. The durations between birth and Exam 1, Exam 1 and Exam 2, and birth and Exam 3 were also documented. The characteristics of the retinal hemorrhages, including severity and location, were investigated using the fundus photographs obtained in Exam 1 and documented.

The severity of hemorrhages was categorized as follows: Grade 0, no hemorrhage; Grade 1, small and relatively few hemorrhages, with the majority having sizes less than one-quarter of the diameter of the optic disc in one or both eyes; Grade 2, larger hemorrhages that do not exceed the diameter of the optic disc or a combination of several small and a few large hemorrhages; Grade 3, hemorrhages larger than the size of the optic disc, which are often extremely large and are several times the diameter of the optic disc [9].

**Figure 1:**
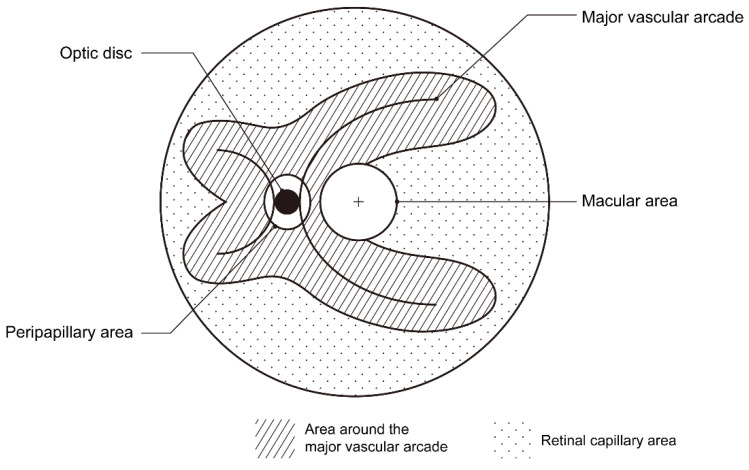
A diagram of the area around the major vascular arcade (MVA) and retinal capillary area (RCA). The MVA area encompassed approximately 1–2 disc diameters around the visible retinal artery and vein, while the other area constituted the RCA.

**Figure 2:**
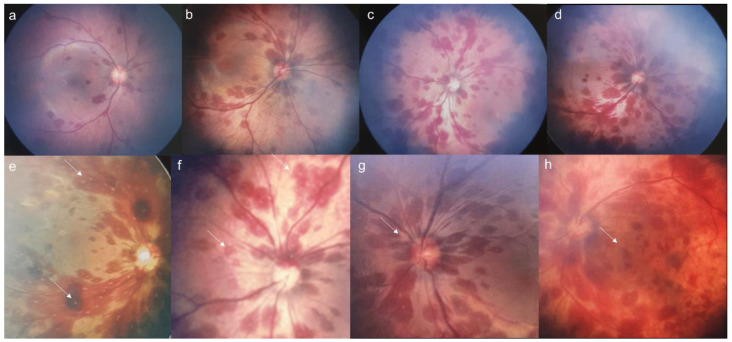
The upper row demonstrates the increase in hemorrhages as the grade becomes more severe. Retinal hemorrhages were shown only around the MVA in grade 1 (**a**) and 2 (**b**). Whitish cores were observed in grade 3 hemorrhages located both in the MVA area and RCA (**c**,**d**). The lower row shows the representative characteristics of retinal hemorrhages. Whitish dots (**e**,**f**), peripapillary hemorrhages (**g**), and macular involvement (**h**) are shown. Arrows indicate the specific characteristics in each image.

The retina was divided into two separate areas: the major vascular arcade (MVA) and retinal capillary area (RCA). The visible retinal arteries and veins constituted the MVA. If the diameter of the visible retinal vessels was over 130–150 μm, they were considered major retinal vessels [13,14,15]. The region encompassing approximately 1–2 disc diameters around the MVA were termed the MVA area. Other areas where large arteries or veins were absent were classified as RCA. Due to the unique pattern of vasculature in each eye, the number of major vascular arcades varied from 3 to 7 (mean 4.5 ± 0.8). A diagram of these two areas is shown in Figure 1, which shows 4 major vascular arcades. Hemorrhages were considered present when at least five large ones were found in the area. Macular and peripapillary involvement were also analyzed. Macular involvement was defined as the presence of at least one large hemorrhage in the macular area: approximately 2 disc diameters area around the fovea. Peripapillary involvement was characterized by a hemorrhage adjacent to the optic disc. The whitish core was characterized by more than 50% of retinal hemorrhages showing visible whitish spots at their centers (Figure 2). All images were independently graded by two ophthalmologists (EJS and GHJ). Disagreements between the two graders were resolved by a third grader (KTK) blinded to the patients’ information. The examiners were blinded to the delivery method of the newborns during the grading process, ensuring that preconceived notions regarding delivery methods did not influence the analysis or interpretation of the data. The inter-rater reliability was high (Cohen’s kappa coefficient = 0.805, *p* < 0.001).

SPSS version 21.0 (SPSS Inc., Chicago, IL, USA) was used for all statistical analyses. Cohen’s kappa coefficients were calculated from the cross-tabulation analysis and averaged. The chi-squared test was used to determine the relationship between retinal hemorrhages and the mode of delivery. Fisher’s exact test was used to determine the relationship between the grade and location/characteristics of the hemorrhages. Student’s *t*-test was used to compare the demographic data, refractive errors between groups, and characteristics of hemorrhages. For all analyses, *p* < 0.05 denoted statistical significance.

## 3. Results

A total of 98 newborns with retinal hemorrhages in at least one eye, and 30 newborns without any retinal hemorrhages in both eyes, were eligible for enrollment. Their demographic data are presented in Table 1. Newborns delivered through NSVD rather than cesarean section were significantly increased in the retinal hemorrhage group compared to the control group (*p* = 0.004). For most cases, the retinal hemorrhages were bilateral (87.8%), whereas unilateral hemorrhages were observed in 12.2% of cases. The newborns in the retinal hemorrhage group underwent Exam 1 using fundus photography after 1.0 ± 0.8 days (range, 1–5 days) from birth and were referred to retinal specialists. After 45.6 ± 15.9 days from Exam 1, Exam 2 using indirect ophthalmoscopy was performed by retinal specialists. All retinal hemorrhages (100%) had resolved at this time.

The distribution of the retinal hemorrhage grades is presented in Table 2. Most neonatal retinal hemorrhages were classified as Grade 2. For most of the newborns (84.7%), both eyes had the same grade or a one-grade difference. Neonates with unilateral hemorrhages were more associated with cesarean section (25.0%) delivery than infants with bilateral hemorrhage (7.0%), with marginal significance (*p* = 0.052). The characteristics and locations of the neonatal hemorrhages are presented in Table 2.

**Figure 3:**
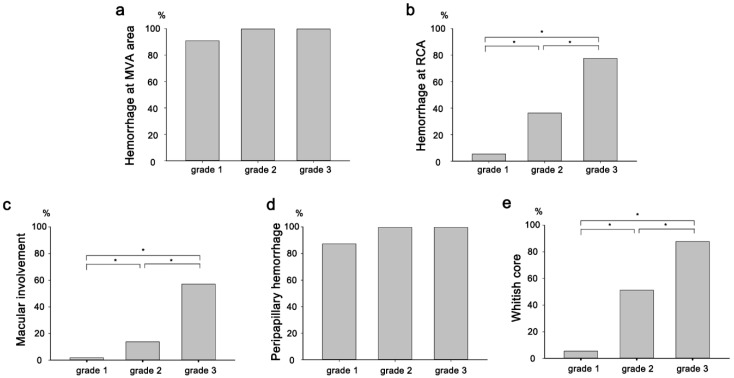
Hemorrhages around the major vascular arcade (MVA) were frequently observed regardless of the grade, while those in the retinal capillary area (RCA) predominantly had higher grades (**a**,**b**). Peripapillary hemorrhages can be observed in most eyes, while macular involvement is associated with high-grade hemorrhages (**c**,**d**). The whitish core appeared related to the hemorrhage grade (**e**). Asterisks (*) indicate a statistically significant difference (*p* < 0.05).

The relationships between the grades, characteristics, and locations of the hemorrhages were investigated (Figure 3). Hemorrhages were frequently observed around the MVA regardless of grade. In contrast, hemorrhages in the RCA increased with higher hemorrhage grades (*p* < 0.001). Similarly, peripapillary involvement was observed in 87.3% of grade 1 hemorrhages and 100% of grade 2 and 3 hemorrhages, while macular involvement and the frequency of the white core increased with the severity of the hemorrhages (*p* < 0.001).

Exam 3, including the cover/uncover test, alternate cover test, and manifest refraction, was performed in 234.5 ± 39.3 days and 231.9 ± 32.0 days in the retinal hemorrhage group and control group, respectively. All the eyes were orthotropic without strabismus in the cover/uncover test and alternate cover test. The mean refractive error was not significantly different between both groups (Table 3). There was no correlation of spherical equivalents to the grade of hemorrhages (*p* = 0.766): +0.55 ± 0.62 D for grade 1, +0.47 ± 0.73 D for grade 2, and +0.65 ± 0.76 D for grade 3. There were also no statistically significant relationships between the refractive errors associated with macular involvement, whitish core characteristics, or retinal capillary area involvement.

## 4. Discussion

We observed retinal hemorrhages at a mean of 1.0 ± 0.8 days after birth with a predominance of a moderate degree, consistent with a previous report [1,5,16,17,18]. No eyes presented with retinal hemorrhages 45.6 ± 15.9 days after the first examination. Previous prospective studies have reported the resolution of retinal hemorrhages within two weeks for the majority of cases [1,4]. Therefore, newborns with persistent retinal hemorrhages for 1–2 months after birth require comprehensive examinations, including hematologic or neurologic tests. The risk of shaken baby syndrome should also be considered.

Several previous studies have reported that the mode of delivery is significantly associated with retinal hemorrhages [1,4,16,19]. Delivery with forceps or vacuum increased the incidence of retinal hemorrhages, while cesarean section decreased it relative to NSVD. The odds of RH were 20.6 times higher with vaginal delivery than with cesarean delivery [20,21]. We also found delivery via cesarean section was correlated with not developing retinal hemorrhage (*p* = 0.004). In infants with retinal hemorrhage, unilateral hemorrhages in newborns were associated with cesarean section delivery, while bilateral hemorrhages were related to NSVD, with marginal significance (*p* = 0.052). It has been hypothesized that a constant suction force of the chignon can cause edematous changes in the brain tissue, resulting in an increase in intracranial pressure, stasis of the central retinal vein flow, and an increase in retinal capillary pressure with retinal hemorrhages [5]. Intracranial pressure can also be increased by simple luminal pressure of the vaginal canal during NSVD. In contrast, cesarean section skips the vaginal passage and prevents the elevation of intracranial pressure. Other maternal and neonatal factors include low birth weight, prolonged labor, instrumental delivery, maternal age, and parity. Neonates with lower birth weights are more prone to RH, possibly due to weaker retinal vessel integrity [21]. Prolonged labor and instrumental delivery increase mechanical stress during the birthing process, leading to higher incidences of hemorrhage [5,20]. Regarding labor duration, several studies have highlighted the significant association between the duration of labor, particularly the second stage, and the incidence of neonatal retinal hemorrhage [22,23]. Prolonged or difficult labor is believed to increase intraocular and intracranial pressures, which may predispose neonates to microvascular injury within the retina [23]. The mechanical stress during prolonged labor can lead to venous congestion and retinal capillary rupture, particularly in the peripapillary and macular regions. Instrumental deliveries, such as forceps or vacuum extraction, further elevate this risk by introducing additional traction forces on the fetal head and ocular structures [24]. Advanced maternal age and primiparity have also been linked to increased RH risk [5]. Additionally, neonatal conditions such as perinatal asphyxia and abnormal coagulation profiles may contribute to the likelihood and severity of RH [25].

The predominance of hemorrhages around the large vessels and optic disc in the present study (90.3% and 91.8%, respectively) also suggests that hemorrhages are associated with an increase in retinal venous flow. Hemorrhages were less frequently observed in the RCA or avascular (macular) area, where the distal end of the retinal vascular system and the propagation of intraluminal pressure were minimal. However, the prevalence of hemorrhages in these areas increased with increasing severity (Figure 3b,e), which indicated that the intracranial or central retinal venous pressure was high.

The precise mechanisms underlying retinal hemorrhages in newborns involve a combination of mechanical, vascular, and hemodynamic factors. During vaginal delivery, particularly under NSVD conditions, elevated intracranial pressure transmitted through the optic nerve sheath plays a significant role. A computational finite element analysis study has shown that the pressure generated during uterine contractions increases intraocular pressure and stresses within the retina and optic nerve sheath [20]. These forces are highest near the optic disc and posterior pole, explaining the predilection for hemorrhages in these areas [20]. Increased intracranial pressure may also obstruct venous outflow through the central retinal vein, leading to the rupture of fragile neonatal retinal vessels. The immaturity of the neonatal microvasculature, characterized by shorter vessel segments, increased branching, and reduced tensile strength, exacerbates vulnerability to mechanical stress [5,26]. The deeper layers of the retina, particularly the intraretinal and subretinal zones, are more susceptible to injury due to their proximity to the elevated pressure zones [26].

Whitish core retinal hemorrhages were observed in 44.4% of the eyes in this study, which was consistent with a previous report [1]. Whitish core retinal hemorrhages can co-occur with systemic illness, such as bacterial endocarditis or other infective diseases, and are also known as Roth’s spots [27]. However, whitish core retinal hemorrhages can also be found in focal ischemia, inflammatory infiltrates, or the accumulation of fibrin, platelets, or neoplastic cells [28,29]. The increased incidence of whitish cores in high-grade retinal hemorrhages indicates that this phenomenon is related to relatively severe vascular damage.

Macular hemorrhage obstructing the visual axis during the critical period can cause deprivation amblyopia [30]. We measured the refractive errors and examined the eyes for strabismus to estimate visual function because detecting amblyopia in infants is challenging. However, compared to the control infants, we could not find significant refractive error changes or strabismus development in infants with retinal hemorrhage in at least one eye. The grade, location, and characteristics of the hemorrhages, even macular involvement, did not alter refractive error change or strabismus development in 7.8 months of follow-up. The macula plays a critical role in high-resolution visual acuity, and any structural disruption during early postnatal periods, a time of rapid visual development, could interfere with normal visual maturation. While the present study found no significant refractive errors or strabismus associated with macular involvement at 7.8 months of follow-up, it remains possible that subtle effects on fine visual function, such as stereopsis or contrast sensitivity, may manifest later in childhood. Incorporating optical coherence tomography into future studies could offer more detailed insights into the resolution dynamics of macular hemorrhages and their potential impact on foveal development. Such imaging could help identify early markers of structural abnormalities, such as disruption of the ellipsoid zone or foveal hypoplasia, which may predispose patients to amblyopia.

Cycloplegic refraction is a standard method for evaluating refractive errors in children, but its use in infants aged six months or younger has been limited due to the reported adverse effects of topical atropine sulfate and cyclopentolate hydrochloride. Systemic absorption of these agents can cause side effects, such as flushing and fever with atropine and drowsiness with cyclopentolate. Moreover, cardiac dysrhythmias have been reported as a major adverse reaction, and topical atropine has been shown to increase heart rate in healthy dogs [12,31]. An alternative approach is noncycloplegic refraction, which has been shown to be effective for measuring refractive errors in most healthy, nonstrabismic infants with a mean age of 5.71 months [32]. Additionally, non-cycloplegic refraction testing has been shown to be effective in screening for abnormal levels of hyperopia in children and neonates [33,34]. These findings suggest that noncycloplegic manifest refraction may be a safer and effective alternative to cycloplegic refraction in this population.

The present study has some limitations. Besides its retrospective nature, only newborns with retinal hemorrhage in the posterior pole were included because the diagnosis was based on fundus photographs, which could not reveal the peripheral retina. The included newborns were born in a local obstetric clinic and referred to the ophthalmology clinic for retinal hemorrhages; therefore, perinatal history (including the Apgar score, meconium aspiration, or birth asphyxia) and maternal information (such as parity or age) could not be collected. Additionally, detailed perinatal conditions, such as neonatal coagulation profiles or specific delivery complications, were not available, which limits our ability to fully analyze their potential influence on retinal hemorrhages. While all included newborns had a history of uneventful deliveries, future studies should aim to incorporate comprehensive perinatal data to better elucidate these relationships. Lastly, the follow-up period is insufficient for detecting refractive change or strabismus development from amblyopia. Longer follow-up until school-age can give sufficient data for determining the relationship between neonatal retinal hemorrhage and amblyopia development.

In conclusion, fundus photographs of neonatal retinal hemorrhages confirmed that the hemorrhages were frequently observed around the large vessels rather than in the capillary area or macula, supporting the hypothesis that the etiology of these hemorrhages is related to elevated intracranial and central retinal venous pressure during delivery. The presence of whitish core hemorrhages indicates a severe grade of vascular damage. Despite the presence of these hemorrhages, macular involvement did not affect refractive error or strabismus development during the 7.8-month follow-up period. However, further investigations are needed to assess their effects on long-term visual function.

## Figures and Tables

**Table 1:** The demographic features of the study participant population.

	Retinal Hemorrhage	Control	*p* Value
**Total number of infants**	98	30	
**Sex (male/female)**	57/41	20/10	0.523 *
**Gestational age (weeks)**	39.3 ± 1.1	39.6 ± 1.3	0.628 ^†^
**Birth weight (kg)**	3.29 ± 0.39	3.36 ± 0.34	0.344 ^†^
**Method of delivery (infants)**			0.004 *
*NSVD*	90 (91.8%)	21 (70.0%)	
*Cesarean section*	8 (8.2%)	9 (30.0%)	
**Laterality (infants)**			
*Bilateral*	86 (87.8%)		
*Unilateral*	12 (12.2%)		
**Time intervals (days)**			
*Birth–Exam 1*	1.0 ± 0.8 (range 1–5)	1.1 ± 0.7 (range 1–5)	
*Exam 1–Exam 2*	45.6 ± 15.9		
*Birth–Exam 3*	234.5 ± 39.3	231.9 ± 32.0	0.841 ^†^
**Refractive errors in Exam 3 (D)**	0.53 ± 0.70	0.69 ± 1.34	0.538 ^†^

NSVD, normal spontaneous vaginal delivery; Exam 1, fundus photography; Exam 2, fundus re-examination with indirect ophthalmoscopy by a retinal specialist; Exam 3, cover/uncover test, alternate cover test, and manifest refraction. * *p* values were calculated with chi-square analysis. ^†^ *p* values were calculated with Student’s *t*-test.

**Table 2:** Grade, characteristics, and location of neonatal retinal hemorrhages.

Total Number of Eyes	196
**Grade (eyes)**	
Grade 0 *	12 (6.1%)
*Grade 1*	55 (28.1%)
*Grade 2*	80 (40.8%)
*Grade 3*	49 (25.0%)
**Grade difference between both eyes (newborns)**	
*0 (same grade)*	38 (38.8%)
*1*	45 (45.9%)
*2*	14 (14.3%)
*3*	1 (1.0%)
**Characteristics (eyes)**	
*Whitish core*	87 (44.4%)
*Macular involvement*	40 (20.4%)
*Peripapillary hemorrhage*	177 (90.3%)
**Location (eyes)**	
*Around the major vascular arcades*	180 (91.8%)
*In retinal capillary area*	70 (35.7%)

* Grade 0 in the study group indicates that the hemorrhage was unilateral.

**Table 3:** A comparison of the refractive errors associated with retinal hemorrhages and their characteristics and locations.

	Present	Absent	*p*-Value
**Retinal hemorrhage (D)**	+0.53 ± 0.70 (184)	+0.60 ± 0.78 (12)	0.787
**Macular involvement (D)**	+0.48 ± 0.53 (40)	+0.55 ± 0.75 (156)	0.679
**Whitish core (D)**	+0.47 ± 0.71 (87)	+0.58 ± 0.70 (109)	0.411
**Retinal capillary area hemorrhage (D)**	+0.49 ± 0.79 (70)	+0.56 ± 0.66 (126)	0.627

D, diopter. The number of eyes is indicated in parentheses.

## Data Availability

The raw data supporting the conclusions of this article will be made available by the authors on request.
